# Leaders of Peer Groups in Chinese Early Adolescents: The Roles of Social, Academic, and Psychological Characteristics in Group Leadership

**DOI:** 10.1007/s10964-024-02003-9

**Published:** 2024-05-15

**Authors:** Jiaxi Zhou, Xinyin Chen, Dan Li, Junsheng Liu, Liying Cui

**Affiliations:** 1 Graduate School of Education, University of Pennsylvania, Philadelphia, PA USA; 2 Department of Psychology, Shanghai Normal University, Shanghai, China; 3 School of Psychology and Cognitive Science, East China Normal University, Shanghai, China

**Keywords:** Peer group leaders, Social and academic competence, Chinese early adolescents

## Abstract

Leadership in peer groups is an important issue in adolescent socioemotional development, yet it has received limited attention in research. This one-year longitudinal study examined peer group leadership and the roles of social, academic, and psychological characteristics in the dynamics of group leadership. Participants included 1061 Chinese students (initial *mean* age =11.17 years; *SD* = 6.98 months; 49.4% female). Data were collected from peer assessments, teacher ratings, and self-reports. The longitudinal social network analysis (SIENA) indicated that peer group leadership was fluid with leadership status evolving over time across groups in a hierarchical manner. Adolescents displaying higher social competence and aggression and lower shyness were more likely to become group leaders. Academic performance and loneliness were not significantly associated with the dynamics of peer group leadership. The results help understand peer group leadership and contributions of social behaviors to the attainment of leadership status in peer groups in early adolescence.

## Introduction

Late childhood and early adolescence represent an important period for the development of leadership skills (Brummelman et al., 2021). The experience of leadership during this period plays a significant role in preparing young individuals for future success (Tackett et al., 2023). Research on leadership in adolescents has focused on formal settings such as classrooms and schools (French et al., 2022). Leadership within informal peer groups, which serves as a microsystem of youth’s daily interactions (Chen, 2023), remains underexplored. The current study aims to fill this gap by examining the dynamics of leadership in peer groups and its relations with social, academic, and psychological attributes among early adolescents.

### Peer Group Leadership

Peer groups serve as a primary setting for children and adolescents to learn social skills, gain support, and build their self-identity through activities guided by group norms (Kindermann & Gest, 2018). In these groups, leaders naturally emerge, tasked with organizing activities, promoting cooperation, navigating challenges, and resolving conflicts (French et al., 2011). Leadership roles within the intimate circles of peer groups may differ from those in broader school or classroom context. For example, adolescents may hold a leadership position within their peer groups without being recognized as a leader or being seen as an effective leader at the class or school level by classmates (Gest et al., 2011), particularly if the peer group has low status or low visibility in school. And, adolescents who are leaders or possess leadership skills in the classroom or school may not necessarily be affiliated with a peer group and be a leader in the group (Paluck & Shepherd, 2012).

Adolescent peer groups are typically formed based on common interests and experiences (Rubin et al., 2015). Leadership within these groups, which refers to the capacity to influence and guide others and facilitate group efforts to accomplish shared goals (Tackett et al., 2023), can be dynamic, allowing for the sharing or change of roles among members (Carson et al., 2007). For example, group members may alternate leadership positions during different activities, leading to reciprocity in group leadership. Furthermore, social hierarchy within adolescent peer groups can be layered, with distinct status among members, ranging from central to peripheral positions (Zarbatany et al., 2019). The hierarchy in leadership may appear cross groups. For example, a leader of a group is a member of another group that has a higher-order leader. Research on friendships has shown that adolescents have a tendency to form connections with the friends of their initial friends (Gremmen et al., 2019). A similar tendency in the domain of peer group leadership may be observed in the pattern of transitivity because adolescents may be interested in interacting and establishing connection with their leader’s leader.

To date, only one study directly examined relations between peer group leadership and social-behavioral factors among adolescents in the United States (Lansford et al., 2009). The results showed that peer group leaders were more likely to engage in problem behaviors than nonleader members (Lansford et al., 2009). However, the cross-sectional design of this study precluded inference about the directions in the relations. Thus, further investigation is clearly needed on this issue.

### Peer Group Leadership and its Associations with Social, School, and Psychological Characteristics: a Bi-Strategic Perspective

Leadership in peer groups emerges from a combination of various behaviors and characteristics that enable individuals to forge social alliances, orchestrate activities, provide instrumental and emotional support to others, resolve conflicts, and convey an image that commands visibility and respect (Tackett et al., 2023). In certain situations, leadership may also necessitate exerting a degree of intimidation to consolidate authority (Hartl et al., 2020). It has been argued that leadership approaches may be represented by two categories: (1) prestige, which refers to the demonstration of knowledge and skills to gain respect and admiration, and (2) dominance, which is characterized by enforcing control through force or intimidation to instill fear (Cheng et al., 2013). Similarly, according to the Resource Control Theory (Hawley & Bower, 2018), obtaining and maintaining group leadership can be seen as an effort to control resources through gaining social dominance and power. Individuals may use prosocial-cooperative (e.g., helping, sharing) and coercive (e.g., aggression, threat, intimidation) strategies to acquire and control resources (Cillessen et al., 2014). The bi-strategic view is consistent with the argument about the complex and ambivalent nature of leadership (Van Vugt et al., 2008). Given their extensive peer interactions spanning social, academic, and psychological domains (Liu et al., 2023), the two categories in the leadership framework may be reflected by the behaviors and attributes that are important for achieving the developmental tasks for school-age adolescents (e.g., Masten et al., 2005). Based on the literature (e.g., Ladd et al., 2000), the present study examined social competence, aggression, shyness, academic performance, and loneliness and their associations with peer group leadership in this study. These aspects are likely to be relevant to the dynamics of peer group leadership from the broad bi-strategic perspective.

As the ability to successfully navigate social situations to achieve personal or group goals, social competence is manifested in problem-solving, conflict management, organizing cooperative activities, and protecting others from negative treatment of others (Gresham, 2016). Research has revealed that children and adolescents expect leaders to contribute to the group goal and to be different from peers who pursue their goals at the expense of others (Margoni et al., 2018). It has been argued that people tend to favor leaders who are prosocial and responsible because they are likely to provide group benefits and distribute resources equitably (Price & Van Vugt, 2014). Group leaders are expected to actively interact with others and take on responsibilities in group activities (Van Vugt et al., 2008). They are also expected to be able and willing to share, assist others in need, and provide comfort to others in difficult situations (Van Vugt et al., 2008). Thus, attributes such as caring for others, effective communication and problem-solving skills, and the ability to help group function fit with leadership expectations (e.g., Stavans & Diesendruck, 2021). The expectations about leadership appear similar in peer groups in childhood and adolescence (French et al., 2022). Thus, adolescents with these attributes are likely to obtain recognition and approval for leadership in the group. In addition to prosocial-cooperative behavior, individuals may use aggression and other coercive behaviors to acquire power and status (Hawley & Bower, 2018). Research on aggression and peer group leadership in the class or school context has provided mixed results; whereas some studies indicated that aggressive tendencies were associated with a decrease in leadership status (Yang et al., 2015), others showed that leaders exhibited aggressive behavior or a combined prosocial and aggressive characteristic (French et al., 2022). Unlike formal settings such as the school or classroom, where leaders are often chosen based on collective evaluations of peers and teachers according to school standards, adolescents’ informal peer groups are typically formed spontaneously out of common interests, attitudes, and desires of group members. In this context, group members may view aggressive adolescents as capable of helping them pursue group goals and protect group interests, especially when interacting with “outsiders.” As such, aggressive adolescents may receive support from members to take on the leadership role in the group.

Researchers who study children’s and adolescents’ social functioning are interested in shyness—a characteristic representing individual tendency to “move away” (as opposed to “move along” and “move against”) from the world. Shyness refers to anxious reactivity to challenging social situations (Coplan et al., 2004), derived from conflictual approach and avoidance motivations (Asendorpf, 1991). Shy individuals are interested in social interaction, but their tendency to interact with others is impeded by their internal feelings of fear, anxiety, and lack of self-confidence, which results in the display of vigilant and reticent behaviors in social situations (Coplan et al., 2004). Although shy adolescents are often affiliated with peer groups (Zhao et al., 2016), research has shown that shyness is either non-significantly (Liu et al., 2017) or negatively associated with school leadership (Yang et al., 2015). This aligns with the bi-strategic leadership theory that views social assertiveness as a crucial leadership quality (Hartl et al., 2020). The negative association between shyness and leadership may be particularly evident in informal peer groups, as they are typically hierarchical in nature and group leaders need to engage in frequent interactions with others and are involved in group organization, such as coordinating activities. As social assertiveness may be needed to effectively carry out leadership responsibilities (Anderson & Kilduff, 2009), shy children, who tend to be non-assertive (Rubin et al., 2009), are less likely than others to acquire leadership roles.

Research has shown that academic achievement is an important factor in adolescents’ peer group interactions in school (Rambaran et al., 2017). The value of instrumental assistance with learning and schoolwork is well recognized for strengthening peer relationships and enhancing group cohesion (Wentzel et al., 2018). High-achieving adolescents are often liked within their peer groups, due to their ability to help others with academic tasks. Superior academic performance and provision of support for others on learning are likely to be conducive to the attainment of social status, influence, and leadership. The role of academic achievement in adolescents’ peer group leadership in school settings fits with the prestige approach in the leadership framework, which emphasizes competence and the acquisition of admiration (Cheng et al., 2013).

Displaying effective leadership behaviors, such as active participation in social interactions with peers in and outside of the group and taking initiative in activities, may be affected by individual psychological functioning. As a major indicator of psychological wellbeing, loneliness represents a negative experience about one’s social-relational life or unfulfilled needs for social belonging (e.g., Asher & Paquette, 2003). Individuals who report high loneliness tend to be self-oriented, passive or hesitant in social settings, and unwilling or unable to provide emotional support to others and promote positive emotional communication in the group (Schwartz-Mette et al., 2020), which is likely to undermine the acquisition of prestige and dominance for leadership (Cheng et al., 2013). Research has shown that loneliness in adolescents is negatively associated with prosocial behavior and social impact (Chen et al., 2004). Thus, it seems reasonable to argue that loneliness may harm adolescents’ ability to obtain endorsement for leadership in peer groups.

Finally, according to the homophily theory (Rubin et al., 2015), peer groups often comprise members with similar traits, such as misconduct (Ellis et al., 2012), shyness (Zhao et al., 2016), academic performance, social competence (Liu et al., 2023), and psychological problems (Conway et al., 2011). Group functioning including activities is also largely based on similar interests and behavioral styles (Laursen, 2017). It is plausible that adolescents who exhibit social, school, and psychological characteristics that are similar to those of others in the group are likely to receive support for leadership.

### Peer Group Leadership and Social, School, and Psychological Characteristics in Chinese Early Adolescents

Cultural context may influence peer group leadership dynamics, with certain traits being more or less pronounced in a specific society (French et al., 2022). For example, in Chinese society where collectivism and group cohesion are highly valued (Chen & French, 2008), group success may be more emphasized than individual benefits in the dynamics of peer group leadership (Carson et al., 2007). Confucian principles, which endorse social hierarchies, may affect Chinese adolescents’ leadership perceptions, fostering a respectful adherence to the established structures (French et al., 2022). In this context, hierarchy in group leadership may be emphasized and disruption of the established order may be highly discouraged.

In Chinese society, leaders are expected to possess virtuous and compassionate qualities and be capable and motivated to protect and care for group members, as leaders have the obligation to serve others (Ma & Tsui, 2015). Thus, like their Western counterparts, Chinese adolescents are inclined to select leaders who display high social competence to help organize cooperative activities and maintain harmony and cohesion in the group. Aggression is generally discouraged in Chinese society due to its potential threat to group harmony (Chen & French, 2008). However, research results indicated that aggression was positively associated with leadership in the class and the school among Chinese adolescents (e.g., French et al., 2022). It is possible that aggression is viewed as an indication of social assertiveness, which is a major attribute of leadership, and that peers tend to select assertive members for the leadership role. In addition, academic achievement has been highly valued as one of the most important tasks for school-age children and adolescents in China (Fu et al., 2020). This context may make the role of academic achievement salient in shaping peer group leadership. High academic achievement is not only an indication of individual success but also a valuable resource for the group (Liu et al., 2023). Group leaders who are academically competent can assist others in the group with academic work and help enhance the reputation and visibility of the group in school, creating a favorable environment for group activities. Thus, it is perceivable that students who perform well academically are likely to be selected as leaders of peer groups.

Over the past several decades, China has changed toward a competitive market-oriented society in which social assertiveness and self-expression are required for success and thus increasingly appreciated (Cai et al., 2020). The social change may have particularly evident implications for social attitudes toward shyness and psychological or emotional wellbeing (Chen, 2019). Specifically, wary, vigilant, and restrained behaviors that shy children and adolescents display in social situations have traditionally been viewed as indications of social maturity and accomplishment in Chinese society, helping shy children obtain social approval and support (Liu et al., 2017). However, due to its impediment to initiative-taking and exploration, shyness in adolescents has become maladaptive and associated with difficulties in peer relationships and adjustment in recent years, which is similar to what has been found in the West (Chen, 2019). At the same time, adolescents are encouraged to develop self-confidence and engage in active social interaction in the new environment (Chen, 2023). Displaying internalizing symptoms including loneliness is regarded as socially incompetent (Liu et al., 2023). Given this background, it seems reasonable to expect that adolescents with high levels of shyness and loneliness are less likely than others to be peer group leaders in today’s China.

## Current Study

Little is known about leadership in adolescent peer groups. The primary purpose of this one-year longitudinal study was to examine the features of peer group leadership and its associations with social, school, and psychological characteristics in Chinese early adolescents using social network analysis (SIENA). Participants were asked to identify their group members and the leader of the group which they were affiliated with. Data on social competence, aggression, shyness, academic performance, and loneliness were collected through classroom-based peer assessments, teacher ratings, and self-reports. The same data were collected in a follow-up study one year later. The study design and network analysis enabled the tracking of changes in peer group leadership and social, academic, and psychological characteristics associated with the changes over time. Based on previous discussion, a reciprocity effect was expected (Hypothesis 1), indicating that nonleader members would be reciprocated with nominations of leadership roles by their leaders. A positive transitivity effect, indicating a higher likelihood of adolescents following the leader of their leader, and a negative three-cycle effect, indicating a lower propensity for adolescents to lead someone who is a leader of their own leader, were expected (Hypothesis 2). Regarding the selection effects, it was expected that adolescents with higher levels of social competence, aggression, and academic performance would be more likely to emerge as leaders in their peer groups, as indicated by positive alter effects for social competence, aggression, and academic performance (Hypothesis 3). Moreover, it was hypothesized that adolescents with higher levels of shyness and loneliness would be less likely to emerge as leaders in their peer groups, as indicated by negative alter effects for shyness and loneliness (Hypothesis 4). Finally, ego effects and ego-alter interactions were considered in the analyses to examine adolescents’ tendency to form leadership relationship with peers who exhibited similar characteristics. It was hypothesized that adolescents would be more likely to establish leadership relationships with peers with more similar attributes, as indicated by positive ego-alter effects of social competence, aggression, shyness, academic performance, and loneliness (Hypothesis 5).

## Methods

### Participants

The participants were 1061 early adolescents (537 boys) initially in the fifth grade in public elementary schools in China. The schools were in a region, consisting mostly of towns, small cities, and surrounding areas, which is home to a population of approximately 2.7 million people. The participants were drawn from 18 classes in 5 regular public elementary schools, with each class consisting of about 50 students. The average age of the students was 11 years and 2 months (*SD* = 6.98 months). The schools were regular public schools that served students in their geographic area, and students came from the residential area near the schools. As required by China’s Ministry of Education, the core curriculum, including Chinese, mathematics, and English, is identical across the country. The structure and organization of the schools are also similar. A head teacher is assigned to each class and is often responsible for instructing one major subject and managing the day-to-day class activities. Thus, the head teacher is typically familiar with the students in the class. In Chinese schools, students are not permitted to switch classes and they follow similar schedules of courses and activities. They are encouraged to take part in social and academic activities in school, which provides extensive opportunities for them to interact with each other.

The majority of the adolescents in this study came from families with low to middle socioeconomic status backgrounds. In the sample, 83.1% of the mothers and 77.7% of the fathers received an education of junior high school or below, 12.2% of the mothers and 16.7% of the fathers received an education of high school, and 4.7% of the mothers and 5.6% of the fathers received an education above high school. Most participants (98%) were of the Han ethnic nationality, the predominant ethnic group in China, making up over 90% of the population.

From the original sample of 1061 students, 948 (90%) participated in the follow-up study one year later. The longitudinal social network analysis was conducted with these students who participated in both the original and follow-up studies. No significant differences were found in the variables at Time 1 between students who participated in the follow-up study and those who did not (*t* = −1.613, *p* = 0.109, to *t* = 0.811, *p* = 0.418).

### Procedure

Students from each class completed a set of measures in the classroom during a regular class time. The measures included group leader nominations, a peer assessment measure of social competence, aggression, and shyness, and a self-report measure of loneliness. The head teacher of each class was asked to evaluate the academic performance of the students. The items in the measures were carefully examined by the research team, using a variety of strategies (e.g., repeated discussion in the research group, interviews with children and teachers, and psychometric analysis). The Western-based measures were translated and then back-translated to ensure comparability with the English version. The measures have been used and shown to be reliable, valid, and appropriate in Chinese adolescents (e.g., Chen et al., 2023). The study was approved by the Institutional Review Board at Shanghai Normal University. The researchers contacted all the public schools in the region through the school board and invited them to participate in the study, and all students in the participating schools were invited to participate, without any specific exclusion criteria. The participation rate was approximately 95% each time. Active written assent was obtained from all participants and active written consent was obtained through the school. A group of faculty and graduate students in psychology in China administered the measures. Extensive explanations of the procedure were provided, and all questions were answered during administration. No evidence was found that the students had difficulties understanding the procedure or the items in the measures.

### Measures

#### Peer group leadership

Participants were asked to report their peer groups and group leaders using a procedure adapted from the Social Cognitive Map (SCM; Cairns et al., 1991) with the following questions: “Do you often hang out with a group in your class? If yes, who are these people you hang around with?” “If there is a leader (or leaders) in the group, who are they?” Participants were allowed to nominate an unlimited number of their classmates, irrespective of gender, as group members or group leaders. However, participants were instructed to nominate only their most important peer group, with the option to not nominate any groups or leaders if they chose. The SCM procedure has demonstrated validity in identifying observed peer groups in prior research (e.g., Ellis et al., 2012). Responses from all participants were collected and used to construct an adjacency matrix that contained group leader nominations. This matrix captured the relationships of leaders with the nominators as well as other nonleader members within each peer group. For example, if Student A nominated Student B as the leader of a group that includes Students A, B, C, and D, the matrix would represent the relationships not only between A and B but also between C and B and between D and B. Since it is common that adolescents might be a leader in one group and be a nonleader member in another group, all leadership nominations were considered as valid. An unweighted network approach was used in the analyses. Within this matrix, each student (identified in the column) was assigned a value of either 0 or 1 relative to each student in the row. This value indicated whether the participant in each column had been nominated as the group leader of the participant in each row (1 = leader; 0 = nonleader). The nominations were from all group members, but self-nominations for leadership were excluded from the data. If Student A nominated himself or herself as the leader of group ABCD, for example, the relationship of Student A leading Student A was excluded. However, the relationships between B and A, C and A, and D and A were retained. This nomination process and matrix construction were the same for each class at Times 1 and 2. As suggested by others (e.g., Rambaran et al., 2017), students who did not participate in the study were not included in the analyses. Students who participated but did not nominate others or were not nominated by others (isolates) were included in the analyses.

#### Social competence, aggression, and shyness

Participants’ social competence, aggression, and shyness were assessed using a peer assessment measure adapted from *The Revised Class Play* (Masten et al., 1985). The measure included 12 items for social competence (e.g., “Is willing to help others,” “Is polite to others”), 7 items for aggression (e.g., “Too bossy,” “Picks on other kids”), and 4 items for shyness (e.g., “Very shy,” “Feelings get hurt easily”). Nominations received from all classmates were used to compute item scores for each student. The item scores were standardized within the class to adjust for differences in the number of nominators. The variables of social competence, aggression, and shyness were formed based on the corresponding items, with higher scores indicating greater levels of social competence, aggression, or shyness. The measure has been used and shown to be reliable and valid in previous studies with Chinese children and adolescents (e.g., Chen et al., 2023). In the present study, the internal reliabilities of the measures at Times 1 and 2 were 0.93 for social competence, 0.92 and 0.90 for aggression, and 0.72 and 0.71 for shyness, respectively.

#### Academic performance

Participants’ academic performance was assessed by the head teacher in each class on three core subjects in Chinese schools: Chinese, mathematics, and English. The teachers rated each participant’s performance on these subjects on a 5-point scale, ranging from 1 (*poor*) to 5 (*excellent*). The scores on the subjects were significantly correlated (*r* = 0.65 to 0.77, *p* < 0.001) and were averaged and then standardized within each class to control for the teacher’s response style and to allow for appropriate comparisons. This measure has been used and shown to be a valid measure of school academic performance in Chinese children and adolescents (e.g., Chen et al., 2018). The internal reliabilities of teacher-rated academic performance were 0.88 and 0.90 at Times 1 and 2, respectively.

#### Loneliness

Loneliness was measured using a self-report measure (Asher et al., 1984). This measure consisted of 16 statements describing feelings of loneliness (e.g., “I have nobody to talk to,” “I feel lonely”). Participants were asked to respond to these statements on a 5-point scale, ranging from 1 (*not at all true*) to 5 (*always true*). The mean score was computed to form a loneliness variable with higher scores indicating greater loneliness. The measure has been used and shown to be reliable and valid in previous studies with Chinese children and adolescents (Chen et al., 2023). In this study, the internal reliabilities were 0.88 and 0.90 at Times 1 and 2, respectively.

### Data Analytic Plan

#### Social network analysis

The Simulation Investigation for Empirical Network Analysis (SIENA; Snijders et al., 2010) was used to examine the relations between peer group leadership and social, school, and psychological characteristics while considering the structural effects such as reciprocity and transitivity. SIENA is effective in differentiating between selection and influence effects. This study focused on selection effects, examining the influence of social competence, aggression, shyness, academic performance, and loneliness on adolescents’ propensity to assume leadership roles, according to the main hypotheses. SIENA assumes that changes in peer group leadership and individual behaviors over time stem from personal choices influenced by the prevailing network structure and personal traits. It is designed to estimate changes in characteristics and relations between observational points, using data from all network members and their relationships, while accounting for structure-based and attribute-based effects. To achieve precise model estimations, SIENA simulates a range of potential network evolutions between data collection waves, adjusting parameters to align closely with observed data. Following the recommendations of other researchers (e.g., Kornienko et al., 2020), a multigroup method was used, treating each classroom as a separate network, to analyze the data. The analyses were performed using the Rsiena package in R (version 1.3.14; Ripley et al., 2023).

For longitudinal social network analysis, it is recommended that continuous attribute variables be converted into nonnegative integers (Ripley et al., 2023). Based on the recommendation, the scores for social competence, aggression, shyness, academic performance, and loneliness were classified into 10 subcategories (from 1 to 10).

#### Model specification

The present study examined network dynamics, incorporating both structural and attribute-based effects. Basic structural effects, such as leadership reciprocity, and gender effects were controlled in the analyses to enhance the precision of the selection effects. The model includes the following effects: *Rate parameters* control for the rate of change in peer group leadership between two time points. *Density* controls for participants’ tendency to have others as peer group leaders. *Reciprocity* refers to the tendency of nonleader members to be nominated as leaders in the next wave by their initial group leaders (e.g., T1: A→B; T2: B→A, with arrows indicating the relationship from a nonleader to a leader). *Transitive triplets* indicate the extent to which a leader (C) of a group with a nonleader member (B) as a leader of another group including A as a nonleader member at Time 1 is likely to become a leader of a group with A as a nonleader member at Time 2 (e.g., A→B, B→C; T2: A→C). *Three cycles* indicate the extent to which a leader (C) of a group with a nonleader member (B) as a leader of another group including A as a nonleader member at Time 1 becomes a nonleader member of a group in which A is a leader at Time 2 (e.g., T1: T1: A→B, B→C; T2: C→A). *Ego* effects represent the tendency of students scoring higher on specific variables (e.g., gender, social competence) to have group leaders. *Alter* effects represent the likelihood of students scoring higher on specific variables to be group leaders. *Ego × Alter* effects refer to the tendency of students with specific characteristics to be nominated as leaders by group members with similar characteristics. *Same gender* effect represents the tendency to have same-gender leaders in the group.

## Results

### Descriptive Data

Of the 948 students who participated in both the original and follow-up studies, the missing data rates were relatively low, with 3.1% missing for both T1 and T2 in leadership nominations, peer-assessed social competence, aggression, and shyness. Missing rates for academic performance were 11.5% at T1 and 8.1% at T2. For loneliness, the missing rates were 3.6% at both T1 and T2. Little’s MCAR test (Little, 1988) indicated that the missing data were completely at random, χ^2^(72) = 40.06, *p* = 0.999. The SIENA software was used to address the missing data in the analyses.

In the sample, 241 students (25.4%) were reported as leaders at Time 1, and 247 students (26.1%) were reported as leaders at Time 2. Of the students, 739 (78.0%) at Time 1 and 738 (77.8%) at Time 2 were reported as nonleader members led by a leader. From the reports, 37.7% and 39.7% were boys with boy leaders, 53.1% and 45.1% were girls with girl leaders, 6.0% and 10.2% were boys with girl leaders, and 3.2% and 5.0% were girls with boy leaders at Times 1 and 2. Students on average were associated with one peer group leader. The proportion of leader-nonleader relationships that were reciprocated was approximately 8.4% and 7.3% at each time. In examining the dyadic nomination data, the Jaccard index was approximately 0.21, suggesting a low stability in the leadership connections over time (i.e., 21% of leadership relationships were from same nonleader-leader pairs across the time points). At the individual level, the correlation between the counts of peer group leader nominations received at Times 1 and 2 was 0.567 (*p* < 0.05), indicating a moderate continuity in leadership status.

Means and standard deviations of the attribute variables and their correlations are presented in Table 1. The results from the Analysis of Variance (ANOVA) indicated that, at both time points, girls had significantly lower scores on aggression and significantly higher scores on social competence, shyness, and academic performance than boys. The variables were correlated weakly to moderately, indicating that they represented related but distinct aspects of adjustment.

**Table 1:** Descriptive Statistics and Correlations among Attribute Variables

	1.	2.	3.	4.	5.	6.	7.	8.	9.	10
*Time 1*										
1. Social competence										
2. Aggression	0.10^**^									
3. Shyness	0.11^**^	0.05								
4. Academic performance	0.42^***^	−0.10^***^	−0.04							
5. Loneliness	−0.26^***^	0.05	0.10^**^	−0.28^***^						
*Time 2*										
6. Social competence	0.78^***^	0.12^**^	0.09^**^	0.38^***^	−0.25^***^					
7. Aggression	0.07^*^	0.78^***^	0.00	−0.10^***^	0.01	0.08^*^				
8. Shyness	0.12^***^	−0.02	0.57^***^	−0.01	0.04	0.16^***^	−0.02			
9. Academic performance	0.44^***^	−0.09^***^	0.02	0.65^***^	−0.25^***^	0.42^***^	−0.10^**^	0.03		
10. Loneliness	−0.16^***^	0.05	0.11^**^	−0.15^***^	0.50^***^	−0.19^***^	0.06	0.16^***^	−0.22^***^	
Boys Mean (*SD*)	2.40 (0.80)	2.42 (1.43)	2.33 (0.76)	5.03 (1.44)	3.30 (1.54)	2.50 (0.89)	2.61 (1.34)	2.43 (0.76)	5.42 (1.47)	2.95 (1.52)
Girls Mean (*SD*)	2.76 (1.32)	2.02 (0.60)	2.80 (1.31)	5.87 (1.46)	3.13 (1.50)	2.70 (1.34)	2.18 (0.56)	2.80 (1.30)	6.17 (1.36)	2.99 (1.54)
*F*-value	24.56^***^	31.39^*^	43.43^***^	69.27^***^	3.06	10.66^**^	40.11^***^	29.80^***^	56.87^***^	0.11

^*^p< 0.05, ^**^p < 0.01*,* ^***^p < 0.001

The classroom intraclass correlations (ICC) were less than 0.05 for all the variables, indicating no cluster effects for the classroom in the present study (e.g., Hox, 2010). Furthermore, the homogeneity for parameters related to the leadership network effects (density, reciprocity, transitivity effect, and three-cycle effects) was tested using sienaTimeTest function. The results indicated no significant variations in the network structural parameters across classrooms, χ^2^(72) = 92.28, *p* = 0.054, suggesting that the dynamics of leadership networks were consistent across classrooms.

### Social Network Analysis

The results based on Rsiena analysis are presented in Table 2, including the estimates and standard errors for the effects. The estimates can be interpreted as log odds of a student would change their group leader (in case of the network dynamic part of the model). Odds ratios can be obtained by taking the exponential of the parameter estimates. With all parameter convergence t-ratios below 0.1 and overall maximum convergence ratios of 0.262, the model fit indicators confirm satisfactory convergence as per guidelines (Snijders et al., 2010). The results of the goodness-of-fit tests are presented in the Appendix.

**Table 2:** RSiena Results Concerning Effects of Social Competence, Aggression, Shyness, Academic Performance, and Loneliness on Peer Group Leadership

Effects	Est.	*SE*
*Network Effects*		
Density	−2.39^***^	0.06
Reciprocity	1.14^***^	0.13
Transitivity	0.77^***^	0.10
Three-cycles	−1.29^***^	0.30
*Selection Effects*		
Social competence alter	0.12^***^	0.03
Social competence ego	−0.02	0.04
Social competence ego × Social competence alter	−0.02	0.03
Aggression alter	0.09^**^	0.03
Aggression ego	0.04	0.04
Aggression ego × Aggression alter	−0.04	0.06
Shyness alter	−0.13^*^	0.05
Shyness ego	−0.01	0.05
Shyness ego × Shyness alter	0.05	0.06
Academic performance alter	0.05	0.04
Academic performance ego	0.01	0.04
Academic performance ego × Academic performance alter	−0.02	0.03
Loneliness alter	0.01	0.03
Loneliness ego	−0.01	0.04
Loneliness ego × Loneliness alter	−0.01	0.03
Gender alter (0 = boys, 1 = girls)	0.30^***^	0.08
Gender ego (0 = boys, 1 = girls)	0.10	0.09
Same gender	−0.01	0.06

^*^p < 0.05, ^**^p < 0.01*,* ^***^p < 0.001

The reciprocity effect was positive, suggesting that nonleader group members tended to be reported as leaders by their leaders in return. There was a significant and positive transitivity effect, and a significant and negative three-cycles effect. The results indicated that when individual C was a leader of a group in which individual A’s leader was a nonleader member at Time 1, individual C was more likely than others to become a leader of a group in which individual A was a nonleader member at Time 2, while individual A was less likely than others to become a leader of a group in which individual C was a nonleader member at Time 2.

Positive alter effects were found for social competence, with an Odds Ratio (OR) of 1.13 (exp(0.12)), and aggression, with an OR of 1.09 (exp(0.09)), and a negative alter effect was found for shyness, with an OR of 0.88 (exp(−0.13)). The results suggested that adolescents higher on socially competent or aggressive behaviors were more likely (1.13 and 1.09 times, respectively) to be identified as group leaders whereas adolescents higher on shyness were less likely (0.88 times) to be group leaders. There was a significant gender alter effect, with an OR of 1.35 (exp(0.30)), suggesting girls were more likely (1.35 times) than boys to be peer group leaders. The alter effects were not significant for academic performance or loneliness.

Social competence, aggression, shyness, academic performance, loneliness, and gender were not significantly associated with the likelihood of being a nonleader member in a group with a leader, as evidenced by the non-significant ego effects. The non-significant ego and alter interaction effects and same gender effect indicated non-significant leadership relationship between adolescents with similar levels of social competence, aggression, shyness, academic performance, loneliness, or the same gender. The interactions among the variables, such as the interaction between social competence and aggression, were considered in the initial analyses. The findings indicated that the interaction effects were not significant, and thus were not included in the final analyses.

## Discussion

Research on leadership within adolescent peer groups is limited, relative to studies conducted in broader contexts such as school and classroom. This study sought to fill the gap by exploring peer group leadership and its associations with social, school, and psychological characteristics in early adolescents. The study’s design allowed for analysis of leadership within specific peer groups, providing a better understanding of group leadership dynamics. The results indicated that peer group leadership was fluid and hierarchical. The results also showed that adolescents exhibiting higher levels of social competence and aggression and lower levels of shyness were more likely than others to obtain peer group leadership roles, indicating the selective nature of peer group leadership. The findings supported and broadened the bi-strategic perspective (Hawley & Bower, 2018) on the significance of specific social characteristics for attaining leadership status in adolescents’ peer groups in Chinese context.

### Features of Peer Group Leadership

The results first showed low stability during the one-year period in group leader nominations at the dyadic level, as indicated by the Jaccard index, indicating the fluidity of peer group leadership among adolescents. However, when examined at the individual level, the number of leadership nominations received by adolescents showed moderate stability over time. The results indicated that whereas group leaders may be consistently recognized by their peers for their leadership qualities, the specific groups or individuals they lead can change. As adolescents’ peer groups are formed spontaneously out of common interests (Rubin et al., 2015), leadership in the group is likely influenced by specific activities and contexts, which provides opportunities for group members to obtain leadership roles. Relatedly, the results about the positive reciprocity effect suggest that group leaders tend to select nonleader group members as their leaders in return, indicating leadership is often shared among group members (Derue & Ashford, 2010).

The significant positive transitivity effect and the negative three-cycles effect together suggest that the leader of an individual’s leader tends to become the individual’s direct leader later whereas it’s less common for an individual to obtain leadership over his or her leader’s leader. These results indicate a hierarchical pattern of leadership among and within peer groups. In this pattern, a group leader (B) may serve as a bridge linking the nonleader members (A) in his or her group with the higher-ranking leader (C) of a group including B as a member. The dynamics of group leadership may occur in different manners: C may take on B’s leadership role to lead A; A may form a direct bond with C; or B may facilitate a connection between A and C, thereby establishing a pathway for leadership influence from A to C. However, the likelihood of A being a leader in a group including C is low.

In short, the results of the study indicated group leadership dynamics that have implications for early adolescents’ social relationships at the group level. Changes in group leadership may bring about different experiences of group leaders and members in the new context.

### Relations between Peer Group Leadership and Social, School, and Psychological Characteristics in Chinese Early Adolescents

The present study revealed that, after structural effects were controlled, social competence and aggression positively predicted later leadership, whereas shyness negatively predicted later leadership. The positive predictive effect of social competence aligns with the literature indicating that socially competent students, who are often active in peer interaction and willing to contribute towards group goals, provide benefits to their peers, and share resources fairly, are likely to attain leadership status (Price & Van Vugt, 2014). The positive association between aggression and peer group leadership suggests that adolescents may also employ aggression to gain group compliance and acquire leadership roles (Hawley, 2014). Aggressive adolescents may use aggressive tactics to exert power and control within the group whereas group members may view aggressive leaders aiding their group in achieving shared goals by intimidating outsiders to protect group interests (Hawley, 2014). In contrast to social competence and aggression, shyness, characterized by wary and anxious reactivity in challenging social situations (Rubin et al., 2009), appears to impede the attainment of leadership. As indicated earlier, although shyness was traditionally valued and encouraged in Chinese society, it has been increasingly regarded as maladaptive with the recent social change that emphasizes individual assertiveness and self-confidence (Chen, 2023). In peer group context, shy adolescents may be viewed as unable or unwilling to lead group activities, carry out leadership responsibilities, and make contributions to achieve group goals. Consequently, shy adolescents are less likely to be selected as leaders for groups. These arguments were consistent with the dominance and prestige perspective on leadership (Cheng et al., 2013). Dominance, characterized by the use of coercion and intimidation, and prestige, which involves demonstration of competence to earn respect, are two main pathways for individuals to acquire leadership status (Cheng et al., 2013). This perspective is supported by the results concerning the positive effects of social competence and aggression and the negative effects of shyness, which may indicate low levels of dominance and prestige, in predicting later peer group leadership.

The results of the present study showed that the predictive effects of academic performance on peer group leadership were not significant, which was inconsistent with the hypotheses. The academic norms of peer groups were found to play a significant role in shaping group activities and individual behavior and performance in Chinese schools in the late 1990s to early 2000s, suggesting that academic orientation was a salient feature of peer group functioning (Chen et al., 2008). In a recent study, however, it was found that group social competence became more important than group academic competence in predicting individual school and psychological outcomes in Chinese early adolescents (Liu et al., 2023). As China changes toward a competitive market-oriented society, social competence is required to achieve success and is thus increasingly appreciated (Liu et al., 2023). The results of the present study suggest that whereas emphasis on social competence in the new context may enhance its significance for group functioning, the declined salience of academic performance may weaken its role in the attainment of peer group leadership. Given the informal nature of peer groups, it is possible that group members do not prioritize academic work in group activities and do not view academic performance of the leader as a crucial attribute to help achieve group goals.

The results showed a non-significant association between loneliness and peer group leadership. In Chinese society, children’s and adolescents’ psychological wellbeing has traditionally been regarded as unimportant and neglected, which may still play a role in social interactions today (Chen, 2023). Adolescents may be hesitant to openly share their feelings of loneliness with peers. Moreover, when adolescents express their feelings of loneliness, peers may not view them as important in determining their leadership selections. Nevertheless, as psychological wellbeing has been receiving increasing attention in China (Cai et al., 2020), researchers should investigate the relations between loneliness and other psychological problems and group leadership in the future.

The results showed non-significant ego effects of the social, academic, and psychological variables, suggesting that these variables did not significantly predict the likelihood of being associated with peer groups with a leader. In other words, nonleader members within these groups might have diverse characteristics and the specific characteristics of nonleader members might not significantly affect group leadership dynamics. The results also showed non-significant ego and alter interaction effects, suggesting that similarities in social, school, and psychological characteristics were not a major factor in guiding adolescents’ choices of group leaders. Given the findings on the similarity of members in peer groups (Low et al., 2013), it seems to be the case that adolescents use different criteria to select group members and leaders. Considering the significant alter effects that indicate leaders were more socially competent and aggressive and less shy than others and non-significant ego and alter interaction effects, it appears that peer group leaders exhibit distinctive social behaviors and that the selection criteria for leaders may not be affected by group members’ characteristics.

Finally, the results showed that girls were more likely than boys to be group leaders. It is possible that, due to gender stereotypes that girls are more interested in maintaining social relationships (Wang et al., 2023), girls may be more likely than boys to be expected to take leadership roles in their peer groups. This expectation may increase girls’ opportunities to obtain leadership.

### Limitations and Future Directions

Several limitations and weaknesses in the study should be noted. First, the analysis relied on an unweighted network approach to identify leadership links between adolescents. This approach did not consider the strength of leadership relationships, such as the intensity or frequency of leadership interaction. Future studies may use different methods in data collection to allow for analysis of weighted networks.

Second, peer nominations were used in this study to assess adolescents’ peer group leadership, reflecting peers’ subjective perceptions of leadership roles. Researchers should use other assessments, such as observations (e.g., Brummelman et al., 2021), to obtain more objective information about group leadership. For adolescents’ characteristics, data were collected from different sources, including peer assessments, teacher ratings, and self-reports. Each type of data may be prone to different biases, which should be considered in understanding the results. Future studies may also incorporate different assessments in analysis to enhance the robustness of results.

Third, based on the literature (Hawley, 2014), the attainment of peer group leadership was discussed in terms of the processes such as perceptions of group members and interactions between leaders and peers within and outside groups. However, these processes were not directly examined in this study. For example, although peer assessments were used to measure adolescents’ aggression based on the perceptions of classmates, the assessment did not identify specific targets and motivations underlying the behavior. It is unclear whether adolescents used aggression to gain compliance from their group members or to intimidate adolescents outside their peer groups for the benefit of their own groups. Thus, it will be interesting in future research to delve deeper into the social-interactional and social-cognitive processes, perhaps using self-reports and observations.

Fourth, the present study was conducted with early adolescents. The experience of peer group leadership and its relations with social, school, and psychological adjustments are likely to vary across developmental periods. It will be important to investigate peer group leadership in other periods, such as middle childhood and adolescence.

Fifth, contributions of peer group leadership to individual development often occur in larger contexts (e.g., Rubin et al., 2015). It will be interesting to examine how other aspects of peer relationships, such as dyadic relationships within the group, social networks, and norms within the classroom, work together with peer group leadership in contributing to individual development. The present study focused on the dynamics between group leaders and their nonleader group members. Relationships among nonleader members were not examined. A future direction is to conduct multiple network analyses to explore both leadership networks and peer group networks to better understand the social structures of adolescent groups.

Sixth, a limitation of SIENA method used in the present study is its variable-centric focus. Future research may consider person-oriented approaches (e.g., Dong et al., 2023) to investigate the complexities of adolescent peer group leadership profiles.

Finally, the present study focused on Chinese adolescents. The results may help understand peer group leadership and social behaviors contributing to group leadership in general. Nevertheless, the study needs to be replicated in other societies, including Western societies in which social relationships may serve different functions in development (Chen et al., 2018). Despite its limitations, this study makes a substantial contribution to the understanding of peer group leadership among Chinese early adolescents.

## Conclusion

The characteristics of leadership in adolescent peer groups remain underexplored. To fill this gap, the current one-year longitudinal study examined peer group leadership and its relations with individual social, academic, and psychological characteristics in Chinese adolescents. The results showed the fluid and hierarchical features of adolescent peer group leadership. The results also showed that social competence and aggression were significant and positive predictors of the emergence of group leadership whereas shyness significantly reduced the likelihood of becoming a group leader. These results indicated the dynamic nature of peer group leadership and the roles of specific social behaviors. As a practical implication, although the results indicated that social competence and aggression could contribute to the acquisition of leadership roles, teachers and parents should encourage adolescents to use socially competent, rather than aggressive, strategies to seek leadership status because social competence not only enhances their chances of becoming a leader but also helps develop positive relationships in broader settings. At the same time, teachers and parents may work with adolescents to encourage them to pay attention to prosocial and other socially competent attributes when selecting their peer group leaders, which is likely to foster supportive and harmonious interactions in the group. Researchers and professionals should help shy adolescents gain leadership experiences by providing assistance for them to learn how to regulate their wary and anxious behaviors and develop self-confidence in peer group interactions. More broadly, educators and policymakers should promote constructive activities in youth’s peer groups. It may be an effective strategy to design group-based education and intervention programs with the dynamics of group leadership taken into consideration.

### Supplementary Information

Supporting Information

## References

[CR1] Anderson, C., & Kilduff, G. J. (2009). Why do dominant personalities attain influence in face-to-face goups? The competence-signaling effects of trait dominance. *Journal of Personality and Social Psychology*, *96*(2), 491–503. 10.1037/a0014201. 19159145 10.1037/a0014201

[CR2] Asendorpf, J. B. (1991). Development of inhibited children’s coping with unfamiliarity. *Child Development*, *62*, 1460–1474. 1786728 10.2307/1130819

[CR3] Asher, S. R., Hymel, S., & Renshaw, P. D. (1984). Loneliness in children. *Child Development*, *55*, 1456–1464. 10.2307/1130015

[CR61] Asher, S. R., & Paquette, J. A. (2003). Loneliness and Peer Relations in Childhood. *Current Directions in Psychological Science, 12*(3), 75-78. 10.1111/1467-8721.01233.

[CR4] Brummelman, E., Nevicka, B., & O’Brien, J. M. (2021). Narcissism and leadership in children. *Psychological Science*, *32*(3), 354–363. 10.1177/0956797620965536. 33533309 10.1177/0956797620965536

[CR5] Cai, H., Huang, Z., & Lin, L., et al. (2020). The psychological change of the Chinese people over the past half-century: A literature review. *Advances in Psychological Science*, *28*(10), 1599–1688. 10.3724/SP.J.1042.2020.01599. 10.3724/SP.J.1042.2020.01599

[CR6] Cairns, R. B., Gariepy, J. L., & Kindermann, T. (1991). *Identifying social groups in natural settings*. University of North Carolina at Chapel Hill.

[CR7] Carson, J. B., Tesluk, P. E., & Marrone, J. A. (2007). Shared leadership in teams: An investigation of antecedent conditions and performance. *Academy of Management Journal*, *50*(5), 1217–1234. 10.2307/20159921. 10.2307/20159921

[CR8] Chen, X. (2019). Culture and shyness in childhood and adolescence. *New Ideas in Psychology*, *53*, 58–66. 10.1016/j.newideapsych.2018.04.007. 10.1016/j.newideapsych.2018.04.007

[CR9] Chen, X. (2023). *Socialization and Socioemotional Development in Chinese Children*. Cambridge University Press.

[CR10] Chen, X., Chang, L., Liu, H., & He, Y. (2008). Effects of the peer group on the development of social functioning and academic achievement: a longitudinal study in Chinese children. *Child Development*, *79*(2), 235–251. 10.1111/j.1467-8624.2007.01123.x. 18366421 10.1111/j.1467-8624.2007.01123.x

[CR11] Chen, X., & French, D. C. (2008). Children’s social competence in cultural context. *Annual Review of Psychology*, *59*, 591–616. 10.1146/annurev.psych.59.103006.093606. 18154504 10.1146/annurev.psych.59.103006.093606

[CR12] Chen, X., Fu, R., Liu, J., Wang, L., Zarbatany, L., & Ellis, W. (2018). Social sensitivity and social, school, and psychological adjustment among children across contexts. *Developmental Psychology*, *54*(6), 1124–1134. 10.1037/dev0000496. 29504774 10.1037/dev0000496

[CR13] Chen, X., He, Y., De Oliveira, A. M., Lo Coco, A., Zappulla, C., Kaspar, V., Schneider, B., Valdivia, I. A., Tse, H. C. H., & DeSouza, A. (2004). Loneliness and social adaptation in Brazilian, Canadian, Chinese and Italian children: A multi-national comparative study. *Journal of Child Psychology and Psychiatry and Allied Disciplines*, *45*(8), 1373–1384. 10.1111/j.1469-7610.2004.00329.x. 15482498 10.1111/j.1469-7610.2004.00329.x

[CR14] Chen, X., Zhou, J., Li, D., Liu, J., Zhang, M., Zheng, S., & Han, X. (2023). Concern for mianzi: Relations with adjustment in rural and urban Chinese adolescents. *Child Development*, *00*, 1–14. 10.1111/cdev.13964. 10.1111/cdev.13964 37417935

[CR15] Cheng, J. T., Tracy, J. L., Foulsham, T., Kingstone, A., & Henrich, J. (2013). Two ways to the top: Evidence that dominance and prestige are distinct yet viable avenues to social rank and influence. *Journal of Personality and Social Psychology*, *104*(1), 103–125. 10.1037/a0030398. 23163747 10.1037/a0030398

[CR16] Cillessen, A. H. N., Mayeux, L., Ha, T., de Bruyn, E. H., & Lafontana, K. M. (2014). Aggressive effects of prioritizing popularity in early adolescence. *Aggressive Behavior*, *40*(3), 204–213. 10.1002/ab.21518. 24338722 10.1002/ab.21518

[CR17] Conway, C. C., Rancourt, D., Adelman, C. B., Burk, W. J., & Prinstein, M. J. (2011). Depression socialization within friendship groups at the transition to adolescence: The roles of gender and group centrality as moderators of peer influence. *Journal of Abnormal Psychology*, *120*(4), 857–867. 10.1037/a0024779. 21842961 10.1037/a0024779 PMC3353414

[CR18] Coplan, R. J., Prakash, K., O’Neil, K., & Armer, M. (2004). Do you “want” to play? Distinguishing between conflicted shyness and social disinterest in early childhood. *Developmental Psychology*, *40*(2), 244–258. 10.1037/0012-1649.40.2.244. 14979764 10.1037/0012-1649.40.2.244

[CR19] Derue, D., & Ashford, S. (2010). Who will lead and who will follow? A social process of leadership identity construction in organizations. *Academy of Management Review*, *35*(4), 627–647. 10.5465/AMR.2010.53503267. 10.5465/AMR.2010.53503267

[CR20] Dong, Z., Huitsing, G., & Veenstra, R. (2023). Positive and negative leadership in late childhood: Similarities in individual but differences in interpersonal characteristics. *Journal of Youth and Adolescence*, *52*(8), 1620–1631. 10.1007/s10964-023-01798-3. 37306833 10.1007/s10964-023-01798-3 PMC10275811

[CR21] Ellis, W. E., Dumas, T. M., Mahdy, J. C., & Wolfe, D. A. (2012). Observations of adolescent peer group interactions as a function of within- and between-group centrality status. *Journal of Research on Adolescence*, *22*(2), 252–266. 10.1111/j.1532-7795.2011.00777.x. 10.1111/j.1532-7795.2011.00777.x

[CR22] French, D. C., Chen, X., Chung, J., Li, M., Chen, H., & Li, D. (2011). Four children and one toy: Chinese and Canadian children faced with potential conflict over a limited resource. *Child Development*, *82*(3), 830–841. 10.1111/j.1467-8624.2011.01581.x. 21418056 10.1111/j.1467-8624.2011.01581.x

[CR23] French, D. C., Shen, M., & Jin, S. (2022). Are adolescent leaders prosocial and aggressive? *International Journal of Behavioral Development*, *46*(4), 346–357. 10.1177/01650254221100250. 10.1177/01650254221100250

[CR24] Fu, R., Lee, J., Chen, X., & Wang, L. (2020). Academic self-perceptions and academic achievement in Chinese children: A multiwave longitudinal study. *Child Development*, *91*(5), 1718–1732. 10.1111/cdev.13360. 32170870 10.1111/cdev.13360

[CR25] Gest, S. D., Osgood, D. W., Feinberg, M. E., Bierman, K. L., & Moody, J. (2011). Strengthening prevention program theories and evaluations: Contributions from social network analysis. *Prevention Science*, *12*(4), 349–360. 10.1007/s11121-011-0229-2. 21728069 10.1007/s11121-011-0229-2 PMC3222146

[CR26] Gremmen, M. C., Berger, C., Ryan, A. M., Steglich, C. E. G., Veenstra, R., & Dijkstra, J. K. (2019). Adolescents’ friendships, academic achievement, and risk behaviors: Same-behavior and cross-behavior selection and influence processes. *Child Development*, *90*(2), e192–e211. 10.1111/cdev.13045. 29450883 10.1111/cdev.13045

[CR27] Gresham, F. M. (2016). Social skills assessment and intervention for children and youth. *Cambridge Journal of Education*, *46*, 319–332. 10.1080/0305764X.2016.1195788. 10.1080/0305764X.2016.1195788

[CR60] Hartl, A. C., Laursen, B., Cantin, S., & Vitaro, F. (2020). A Test of the Bistrategic Control Hypothesis of Adolescent Popularity. *Child Development*, *91*(3), e635–e648. 10.1111/cdev.13269. 31237358 10.1111/cdev.13269

[CR28] Hawley, P. H. (2014). The duality of human nature: Coercion and prosociality in youths’ hierarchy ascension and social success. *Current Directions in Psychological Science*, *23*(6), 433–438. 10.1177/0963721414548417. 10.1177/0963721414548417

[CR29] Hawley, P. H., & Bower, A. R. (2018). Evolution and peer relations: Considering the functional roles of aggression and prosociality. In W. M. Bukowski, B. Laursen, & K. H. Rubin (Eds), Handbook of peer interactions, relationships, and groups (pp. 106–122). Guilford Publications.

[CR30] Hox, J. J. (2010). Multilevel analysis: Techniques and applications (2nd ed.). Routledge/Taylor & Francis Group.

[CR31] Kindermann, T. A., & Gest, S. D. (2018). The peer group: Linking conceptualizations, theories, and methods. In W. M. Bukowski, B. Laursen, & K. H. Rubin (Eds.), *Handbook of peer interactions, relationships, and groups* (pp. 84–105). Guilford Press.

[CR32] Kornienko, O., Ha, T., & Dishion, T. J. (2020). Dynamic pathways between rejection and antisocial behavior in peer networks: Update and test of confluence model. *Development and Psychopathology*, *32*, 175–188. 10.1017/S0954579418001645. 30722801 10.1017/S0954579418001645 PMC6930972

[CR33] Ladd, G. W., Buhs, E. S., & Seid, M. (2000). Children’s initial sentiments about kindergarten: Is school liking an antecedent of early classroom participation and achievement? *Merrill-Palmer Quarterly*, *46*, 255–279.

[CR34] Lansford, J. E., Costanzo, P. R., Grimes, C., Putallaz, M., Miller, S., & Malone, P. S. (2009). Social network centrality and leadership status: Links with problem behaviors and tests of gender differences. *Merrill-Palmer Quarterly*, *55*(1), 1–25. 19763241 10.1353/mpq.0.0014 PMC2745121

[CR35] Laursen, B. (2017). Making and keeping friends: The importance of being similar. *Child Development Perspectives*, *11*(4), 282–289. 10.1111/cdep.12246. 10.1111/cdep.12246

[CR36] Little, R. J. A. (1988). A test of missing completely at random for multivariate data with missing values. *Journal of the American Statistical Association*, *83*(404), 1198–1202. 10.1080/01621459.1988.10478722

[CR37] Liu, M., Chen, X., Fu, R., Li, D., & Liu, J. (2023). Social, academic, and psychological characteristics of peer groups in Chinese children: Same-domain and cross-domain effects on individual development. *Developmental Psychology*, *59*(1), 57–68. 10.1037/dev0001449. 36048097 10.1037/dev0001449

[CR38] Liu, J., Chen, X., Zhou, Y., Li, D., Fu, R., & Coplan, R. J. (2017). Relations of shyness–sensitivity and unsociability with adjustment in middle childhood and early adolescence in suburban Chinese children. *International Journal of Behavioral Development*, *41*(6), 681–687. 10.1177/0165025416664195. 10.1177/0165025416664195

[CR39] Low, S., Polanin, J. R., & Espelage, D. L. (2013). The role of social networks in physical and relational aggression among young adolescents. *Journal of Youth and Adolescence*, *42*(7), 1078–1089. 10.1007/s10964-013-9933-5. 23504600 10.1007/s10964-013-9933-5

[CR40] Ma, L., & Tsui, A. S. (2015). Traditional Chinese philosophies and contemporary leadership. *Leadership Quarterly*, *26*(1), 13–24. 10.1016/j.leaqua.2014.11.008. 10.1016/j.leaqua.2014.11.008

[CR41] Margoni, F., Baillargeon, R., & Surian, L. (2018). Infants distinguish between leaders and bullies. *Proceedings of the National Academy of Sciences of the United States of America*, *115*(38), E8835–E8843. 10.1073/pnas.1801677115. 30181281 10.1073/pnas.1801677115 PMC6156645

[CR42] Masten, A. S., Morison, P., & Pellegrini, D. S. (1985). A revised class play method of peer assessment. *Developmental Psychology*, *21*, 523–533. 10.1037/0012-1649.21.3.523

[CR43] Masten, A. S., Roisman, G. I., Long, J. D., Burt, K. B., Obradović, J., Riley, J. R., Boelcke-Stennes, K., & Tellegen, A. (2005). Developmental cascades: Linking academic achievement and externalizing and internalizing symptoms over 20 years. *Developmental Psychology*, *41*(5), 733–746. 10.1037/0012-1649.41.5.733. 16173871 10.1037/0012-1649.41.5.733

[CR44] Paluck, E. L., & Shepherd, H. S. (2012). The salience of social referents: A field experiment on collective norms and harassment behavior in a school social network. *Journal of Personality and Social Psychology*, *103*(6), 899–915. 10.1037/a0030015. 22984831 10.1037/a0030015

[CR45] Price, M. E., & Van Vugt, M. (2014). The evolution of leader-follower reciprocity: The theory of service-for-prestige. *Frontiers in Human Neuroscience*, *8*, 1–17. 10.3389/fnhum.2014.00363. 24926244 10.3389/fnhum.2014.00363 PMC4045238

[CR46] Ripley, R. M., Snijders, T. A. B., Boda, Z., Vörös, A., & Preciado, P. (2023). *Manual for RSiena*. University of Oxford, Department of Statistics; Nuffield College.

[CR47] Rambaran, J. A., Schwartz, D., Badaly, D., Hopmeyer, A., Steglich, C., & Veenstra, R. (2017). Academic functioning and peer influences: A short-term longitudinal study of network-behavior dynamics in middle adolescence. *Child Development*, *88*(2), 523–543. 10.1111/cdev.12611. 27580016 10.1111/cdev.12611

[CR48] Rubin, K. H., Bukowski, W., & Bowker, J. C. (2015). Children in peer groups. In M. H. Bornstein & T. Leventhal (Eds.), *Handbook of child psychology and developmental science. Vol. 4: Ecological settings and processes* (pp. 321412). Wiley.

[CR49] Rubin, K. H., Coplan, R., & Bowker, J. (2009). Social withdrawal in childhood. *Annual Review of Psychology*, *60*, 141–171. 18851686 10.1146/annurev.psych.60.110707.163642 PMC3800115

[CR50] Schwartz-Mette, R. A., Shankman, J., Dueweke, A. R., Borowski, S., & Rose, A. J. (2020). Relations of friendship experiences with depressive symptoms and loneliness in childhood and adolescence: A meta-analytic review. *Psychological Bulletin*, *146*(8), 664–700. 10.1037/bul0000239. 32406698 10.1037/bul0000239

[CR51] Snijders, T. A. B., Van de Bunt, G. G., & Steglich, C. E. G. (2010). Introduction to stochastic actor-based models for network dynamics. *Social Networks*, *32*, 44–60. 10.1016/j.socnet.2009.02.004. 10.1016/j.socnet.2009.02.004

[CR52] Stavans, M., & Diesendruck, G. (2021). Children hold leaders primarily responsible, not entitled. *Child Development*, *92*(1), 308–323. 10.1111/cdev.13420. 32725647 10.1111/cdev.13420

[CR53] Tackett, J. L., Reardon, K. W., Fast, N. J., Johnson, L., Kang, S. K., Lang, J. W. B., & Oswald, F. L. (2023). Understanding the leaders of tomorrow: The need to study leadership in adolescence. *Perspectives on Psychological Science*, *18*(4), 829–842. 10.1177/17456916221118536. 36350711 10.1177/17456916221118536

[CR54] Van Vugt, M., Hogan, R., & Kaiser, R. B. (2008). Leadership, followership, and evolution: Some lessons from the past. *American Psychologist*, *63*(3), 182–196. 10.1037/0003-066X.63.3.182. 18377108 10.1037/0003-066X.63.3.182

[CR55] Wang, Z., Zhao, L., Guan, J., & Zuo, G. (2023). The negative effects of positive gender stereotypes: Evidence from a collectivistic cultural context. *Sex Roles*, *89*(11–12), 786–800. 10.1007/s11199-023-01413-6. 10.1007/s11199-023-01413-6

[CR56] Wentzel, K. R., Jablansky, S., & Scalise, N. R. (2018). Do friendships afford academic benefits? A meta-analytic study. *Educational Psychology Review*, *30*(4), 1241–1267. 10.1007/s10648-018-9447-5. 10.1007/s10648-018-9447-5

[CR57] Yang, F., Chen, X., & Wang, L. (2015). Shyness-sensitivity and social, school, and psychological adjustment in urban Chinese children: A four-wave longitudinal study. *Child Development*, *86*(6), 1848–1864. 10.1111/cdev.12414. 26331958 10.1111/cdev.12414

[CR58] Zarbatany, L., Ellis, W. E., Chen, X., Kinal, M., & Boyko, L. (2019). The Moderating role of clique hierarchical organization on resource control by central clique members. *Journal of Youth and Adolescence*, *48*(2), 359–371. 10.1007/s10964-018-0972-9. 30560514 10.1007/s10964-018-0972-9

[CR59] Zhao, S., Chen, X., Ellis, W., & Zarbatany, L. (2016). Affiliation with socially withdrawn groups and children’s social and psychological adjustment. *Journal of Abnormal Child Psychology*, *44*(7), 1279–1290. 10.1007/s10802-015-0120-x. 26712452 10.1007/s10802-015-0120-x

