# Supplementary material for: Leaders of Peer Groups in Chinese Early Adolescents: The Roles of Social, Academic, and Psychological Characteristics in Group Leadership
Source: J Youth Adolesc. 2024 May 15;53(9):2151–64. doi: 10.1007/s10964-024-02003-9 (PMC11333535; doi:10.1007/s10964-024-02003-9)
Supplement: Supplementary file 1 — Supporting Information [file 10964_2024_2003_MOESM1_ESM.docx]

Supporting Information to:

Leaders of Peer Groups in Chinese Early Adolescents: The Roles of Social, Academic, and Psychological Characteristics in Group Leadership

**Goodness of Fit Results for Auxiliary Statistics**

The goodness of fit was assessed using the sienaGOF() function, which involved analyzing the outdegree distribution, indegree distribution, and geodesic distance to evaluate how well the leadership patterns in the network were captured by the included model effects. The fit across all auxiliary statistics was acceptable, as the simulated values closely matched the observed values, with the *p*-value for the Monte Carlo test exceeding .05.

Violin plots were used to examine the distribution of simulated values in relation to the observed values within a five percent margin of error for specific statistics. The observed values are indicated by red solid lines whereas the boxplots and violin diagrams illustrate the simulated values’ distribution. As shown in Figures S1, S2, and S3, the lower range of the indegree/outdegree distribution was underrepresented whereas the middle and higher ranges were overrepresented. The representation of direct connections (geodesic distance 1) and nearby indirect connections (geodesic distance 2) was more accurate compared to distant indirect connections (geodesic distances 3-5).


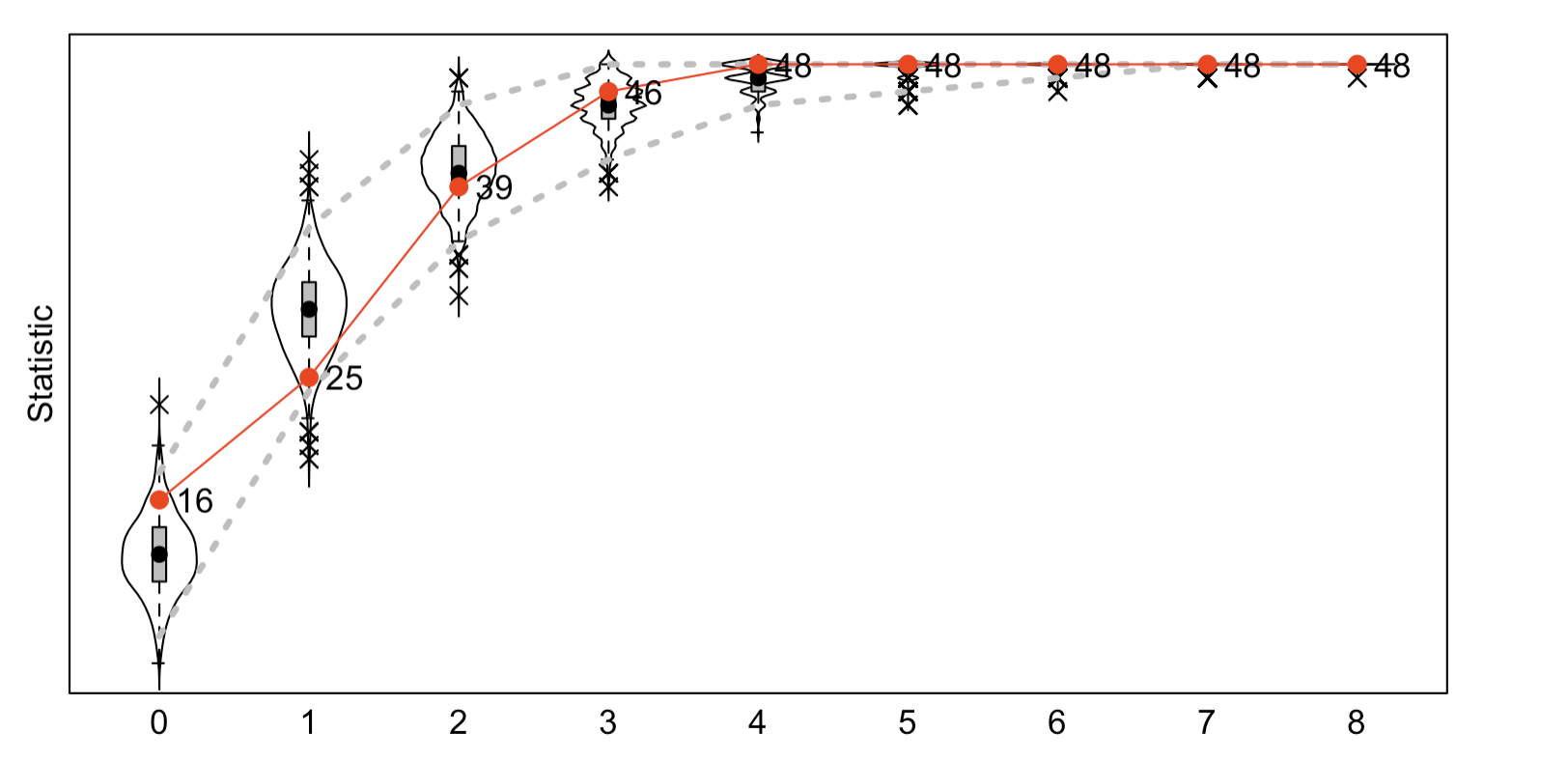


**Figure S1.** Goodness of fit results of outdegree of Model.


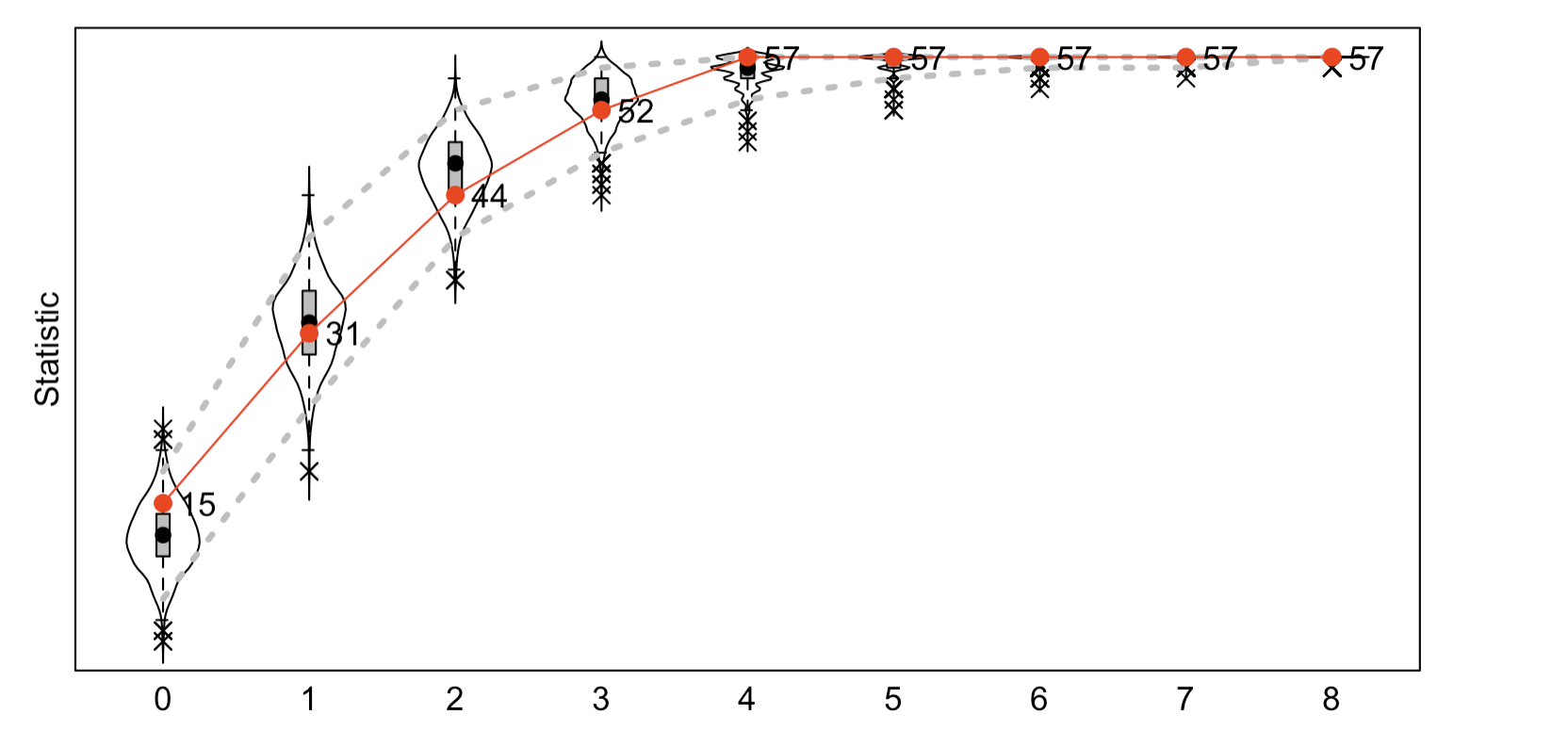


**Figure S2.** Goodness of fit results of indegree of Model.


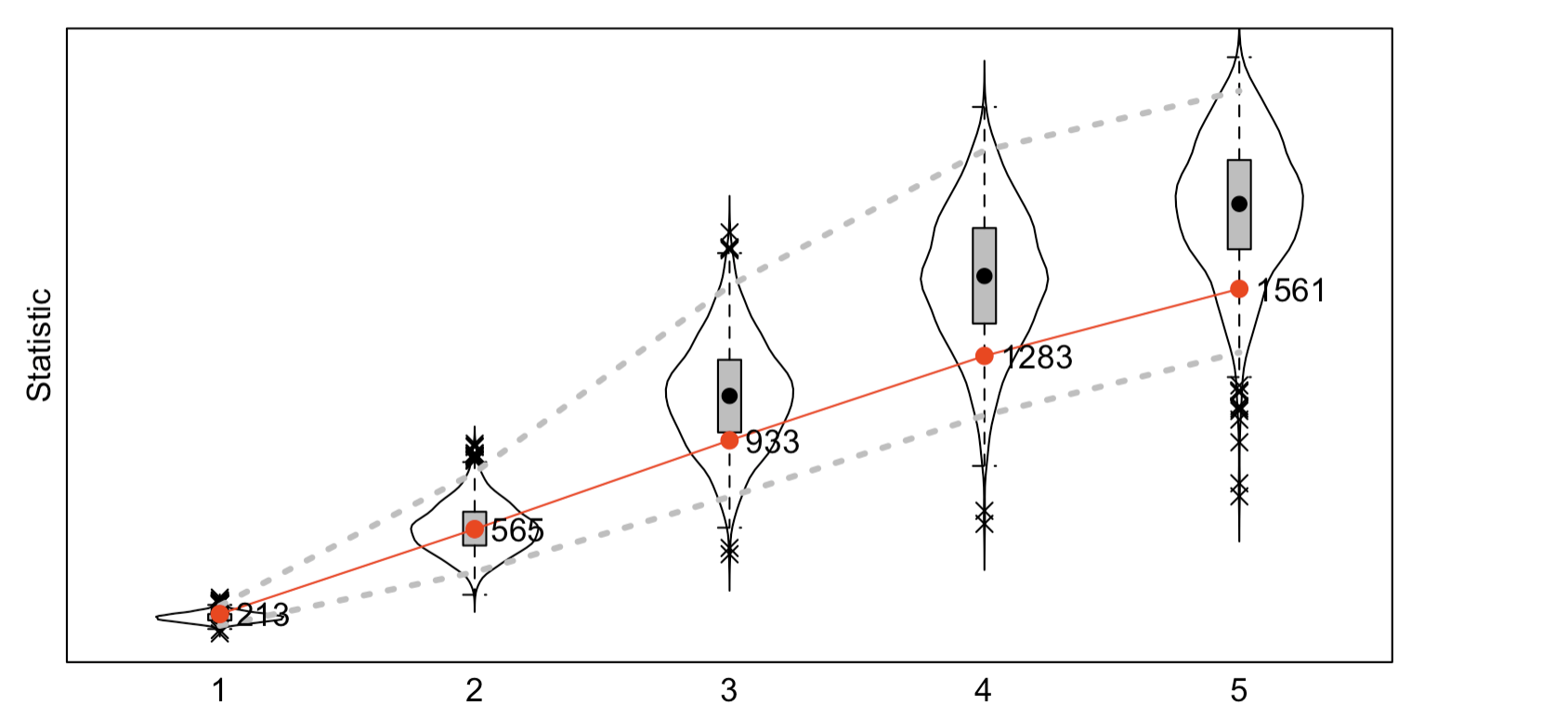


**Figure S3.** Goodness of fit results of geodesic distance of Model.
